# Mortality Trends and Characteristics in a Tertiary Hospital in Southwest Saudi Arabia: A 5-Year Retrospective Study

**DOI:** 10.3390/medicina61081334

**Published:** 2025-07-24

**Authors:** Layla Ali Shaabi, Mohamed Salih Mahfouz, Ahmed Essa Shamakhi, Fathadin Ali Abdu Alahdal, Ali Hakamy, Fatma Rajhi

**Affiliations:** 1 Department of Statistics, King Fahad Central Hospital, Abu Arish 82666, Saudi Arabia; 2 Department of Family and Community Medicine, Faculty of Medicine, Jazan University, Jazan 45142, Saudi Arabia; 3 Department of Pediatrics, King Fahad Central Hospital, Abu Arish 82666, Saudi Arabia; 4 Respiratory Therapy Department, King Fahad Central Hospital, Abu Arish 82666, Saudi Arabia; falahdal@moh.gov.sa; 5 Nursing Department, College of Nursing and Health Sciences, Jazan University, Jazan 82911, Saudi Arabia; aohakamy@jazanu.edu.sa; 6 Research and Studies, Jazan Health Cluster, Ministry of Health, Jazan 86226, Saudi Arabia; frajhi@moh.gov.sa

**Keywords:** hospital mortality, intensive care beds, medical records, length of stay, Jazan and Saudi Arabia

## Abstract

*Background and Objectives:* Hospital mortality rates have repeatedly been used as important indicators of the quality of care provided and as a good monitoring and evaluation tool. Studies on hospital mortality in Saudi Arabia are scant, with most of the available literature focusing on the COVID-19 era. In this study, the patterns and trends in inpatient mortality at King Fahad Central Hospital in southwest Saudi Arabia from 2018 to 2022 were analyzed. Mortality characteristics, including age-specific mortality rates and associated factors, were also investigated. *Materials and Methods:* This was a retrospective study analyzing hospital mortality data in King Fahad Central Hospital (KFCH) from 2018 to 2022 using the largest hospital discharge database in the Jazan region. The mortality rates were calculated, and 95% confidence intervals (CIs) were reported. The analysis also documented some associations using logistic regression models. *Results:* Of the 62,534 patients admitted, 36,971 (59.1%) were females, and 25,543 (40.9%) were males. The mean age (standard deviation) was 24.6 (22.8) years. The overall hospital mortality was 4.8% [95% CI: 4.6–5.0] and was significantly higher among males [7.0%, 95% CI: 6.7–7.3] than females [3.2% 95% CI: 3.1–3.4] (*p* < 0.05). Mortality was significantly higher in the population aged 60 years and above [17.25%, 95% CI: 16.3–18.2] (*p* < 0.001). During the five-year period analyzed, mortality was low in 2018 (3.3%), with remarkably high rates during the COVID-19 period of 2020 and 2021 (5.6% and 6.0%, respectively). The disease groups with the highest prevalence of mortality include certain conditions originating in the perinatal period. In the logistic regression model, the male sex [odds ratio OR = 2.3, 95% CI = 2.01–2.43) was associated with an increased mortality risk. Compared to intensive care beds, general bed departments are associated with a 98% lower risk of mortality [OR = 0.015, 95% CI = 0.014–0.017]. *Conclusions:* This analysis of hospital data statistics revealed a relatively low hospital mortality rate in Jazan. However, the high mortality rates among male patients require further analysis and investigation. Customized interventions targeting high-mortality diseases are recommended.

## 1. Introduction

Hospital mortality rates have frequently been used as indicators of the quality of care provided by healthcare facilities and as a good tool for monitoring and evaluation [1,2,3]. High mortality rates may signal issues with patient safety, treatment effectiveness, or healthcare system deficiencies [4,5], and an important dimension of investigating hospital mortality is identifying areas where patient safety measures need improvement. Using this crucial data, hospitals can implement strategies to reduce medical errors, preventable complications, and adverse events that lead to mortality [6].

Hospitals use mortality data to evaluate the effectiveness of treatments and interventions. By studying mortality rates, healthcare providers can assess the impact of various medical procedures and interventions on patient outcomes. Mortality data are also used by hospitals and healthcare systems to allocate resources effectively. Understanding which conditions or procedures have higher mortality rates can inform resource allocation decisions and healthcare policy development [7].

Policymakers rely on hospital mortality data to make informed decisions about healthcare policy and resource allocation. Thus, data on mortality rates can inform policies related to healthcare financing, accreditation, and quality improvement [8].

Analyzing mortality data is a fundamental part of a hospital’s continuous quality improvement efforts. It allows for healthcare organizations to identify areas for improvement and implement changes to enhance patient care [9].

Assessing hospital mortality is vital for enhancing patient safety, guiding resource allocation, advancing medical research, informing healthcare policies, and empowering patients and their families to make informed decisions about their health [1]. It plays a crucial role in ensuring that healthcare systems provide the best possible care and outcomes for patients [9].

Studies on hospital mortality in Saudi Arabia are extremely limited, and most of the available literature focuses on the COVID-19 era [10,11,12]. Moreover, no studies analyzing hospital mortality in Jazan, southwest Saudi Arabia, have been conducted to date. Hence, the present work focuses on analyzing hospital mortality data in King Fahad Central Hospital (KFCH) during the 2018–2022 period using the largest hospital discharge database in the Jazan region. The aim of this study is to examine the patterns and trends in inpatient mortality and investigate mortality characteristics [age-specific mortality rates, associated causes, length of stay, and other related factors] during this five-year period. This study’s results may contribute to reducing hospital mortality and improving healthcare services. Analyzing mortality cases provides valuable insights into preventable deaths and can provide crucial lessons for international healthcare systems, helping identify best practices, systemic weaknesses, and opportunities for improvement.

## 2. Materials and Methods

### 2.1. Study Design, Data Sources, and Setting

This work was designed as a retrospective observational study using the KFCH Medical Records database for the period from 1 January 2018 to 31 December 2022. The database involves records of 62,534 patients. The study team constructed a checklist to collect the data on the required information, which was then extracted from the hospital’s statistical systems. King Fahd Central Hospital in Jazan (KFCHJ) is one of the largest hospitals located in the Jazan region and has a capacity of 345 inpatient beds. It is accredited by the Saudi Central Board for Accreditation of Healthcare Institutions (CBHAI). In the last five years, the number of beds changed due to both maintenance needs and the COVID-19 pandemic. The hospital offers general surgery, orthopedic surgery, neurosurgery, vascular surgery, cardio-thoracic surgery, urology, plastic and reconstructive surgery, pediatric surgery, maxillofacial surgery, ophthalmology, and otolaryngology (E.N.T). Internal medicine subspecialties include nephrology, gastroenterology, hepatology, pulmonology, rheumatology, dermatology, cardiology, general internal medicine, and E.N.T. Other services include pediatric cardiology, endocrinology, gastroenterology, infectious disease, intensive care medicine, nephrology, neurology, rheumatology, pediatrics, genetics and metabolism, immunology and allergy, neonatology, and obstetrics and gynecology. The hospital also hosts endocrinology, diabetes, and renal disease centers.

### 2.2. Study Variables and Operational Definitions

We considered one main outcome, i.e., mortality rate, defined as death in hospital during index admission and discharge time. The inpatient hospital death rate is the number of inpatients who died in the hospital divided by the total number of hospitalizations. For length of hospital stay, we distinguished long LOS, defined as an LOS greater than the 75th percentile for the specific diagnosis or procedure group (upper-quartile LOS). The demographic characteristics examined included age and sex, while the clinical characteristics included the primary diagnosis and up to six additional sub-diagnoses coded with the International Classification of Diseases, Ninth Revision (ICD-10) as well as the type of admission (emergency or elective). For the purposes of the analysis, age groups were categorized as follows: infants under 1 year; children aged 1–4 and 5–14 years; and patients aged 15–29, 30–44, 45–59, and more than 60 years.

### 2.3. Statistical Analysis

The anonymous data was extracted from the hospital medical record system and then exported into the SPSS computer program ver. 29. Data validation practices were implemented to eliminate the possibility of errors. Continuous variables were summarized using descriptive statistics (mean ± standard deviation [SD]), whereas categorical variables were summarized as frequencies and proportions. Patient characteristics of the deceased and discharged groups were compared using chi-square tests for categorical variables and *t*-tests for continuous variables. The mortality rate was calculated, and the 95% CI was reported. Multiple logistic regression models were used to document associations between the variables. Since we were dealing with a large database of patient indicators, the effect size, based on Cohen’s d and omega squared, was calculated. Cohen’s d was interpreted as follows: <20, very small effect; 0.20–0.49, small effect; 0.51–0.80, intermediate effect; and >0.8, large effect. An omega-squared effect size of 0.01–0.06 was considered small, 0.06–0.14 was considered medium, and >0.14 was considered large. Time series analysis based on the Box–Jenkins approach was utilized to assess mortality trends. A *p*-value < 0.05 was considered statistically significant.

### 2.4. GenAI Use in the Manuscript

During the preparation of this manuscript, we utilized ChatGPT (model: GPT-4) to assist with language editing and rephrasing. The tool was accessed via the following URL: https://chatgpt.com/, accessed on 20 May 2025. All scientific content, data analysis, and conclusions were generated by the authors independently, and any AI-assisted rephrasing was carefully reviewed and approved by the authors to ensure accuracy and integrity.

### 2.5. Ethical Consideration

We obtained ethical approval from the Institutional Review Board (IRB) of the Jazan Health Ethics Committee NCBE-KACST, KSA: H-10-Z-073 (letter no. 22114 dated 19 September 2023). A consent waiver was requested, as the study relied on secondary data. Data were handled following the ethical guidelines of the Helsinki Declaration and the local guidelines of the National Committee of Bioethics, Saudi Arabia.

## 3. Results

Table 1 outlines the demographic characteristics of patients admitted between 2018 and 2022, with a total sample size of 62,534. Females comprised 59.1% (36,971 patients) of the total, while males accounted for 40.9% (25,543 patients). During this period, the proportion of male patients steadily increased from 37.9% in 2018 to 44.3% in 2022, while the percentage of female patients saw a slight decrease. The majority of patients were from younger age groups, with 25.8% (16,128 patients) aged under 1 year, 22.6% (14,156 patients) aged 15–29 years, and 21.9% (13,681 patients) aged 30–44 years. Notably, the trend in age distribution showed that the proportion of patients aged 60 years and older rose significantly from 6.0% in 2018 to 11.5% in 2022, indicating an aging patient population. Additionally, the percentage of patients aged 1–4 years and those aged 5–14 years also experienced modest growth over the years.

Saudi patients represented more than 75% of admissions from 2018 to 2020 and increased to over 80% during the last two years of the study. Most patients were admitted to general beds, totaling 85.6% (53,499 patients). The percentage of patients admitted to the ICU rose from 10.5% in 2018 to 16.8% in 2022, which may indicate changes in the severity of cases (COVID-19). Additionally, the average age of the patients increased from 21.3 years (SD: 20.6) in 2018 to 26.2 years (SD: 23.7) in 2022, suggesting that a larger proportion of older patients were being admitted. Furthermore, the mean length of stay (LOS) decreased from 7.8 days in 2018 to 6.6 days in 2022, indicating improvements in efficiency or shorter hospital stays (Table 1).

Table 2 shows inpatient hospital deaths from 2018 to 2022 based on various characteristics, totaling 62,534 hospitalizations. During this period, there were 2996 total deaths, resulting in an overall mortality rate of 4.8 deaths per 100 hospitalizations, with a 95% confidence interval (CI) of 4.6–5.0 deaths per 100 hospitalizations. The male mortality rate was 7.0 deaths per 100 hospitalizations (95% CI: 6.7–7.3), while the female mortality rate was 3.2 deaths per 100 hospitalizations (95% CI: 3.1–3.4). The difference between these rates was statistically significant (*p* < 0.001), with a small effect size (ϕ = 0.09).

Mortality rates increased significantly with age (*p* < 0.001), exhibiting a moderate effect size (V = 0.21). Additionally, mortality rates rose with longer hospital stays: short stays showed 2.0 deaths per 100 hospitalizations (95% CI: 1.8–2.1), medium stays showed 3.3 deaths per 100 (95% CI: 3.1–3.6), and long stays resulted in 10.3 deaths per 100 (95% CI: 9.9–10.8). This trend was also statistically significant (*p* < 0.001), with a moderate effect size (V = 0.17). When analyzing mortality by department, the intensive care unit (ICU) had a significantly higher rate of 29.4 deaths per 100 hospitalizations (95% CI: 28.5–30.4) compared to general beds, which reported only 0.6 deaths per 100 (95% CI: 0.6–0.7). This difference was highly significant (*p* < 0.001), with a large effect size (V = 0.47). Mortality rates peaked during the years 2020–2021, likely due to the COVID-19 pandemic. This observation is also statistically significant (*p* < 0.001) but has a small effect size (V = 0.05) (Table 2).

Table 3 summarizes inpatient hospital deaths during the study period from 2018 to 2022, categorized by selected variables. Mortality rates peaked in 2020 during the COVID-19 pandemic, with rates of 31.4% in the ICU and 11.8% among long-stay patients. Throughout the entire period, male patients consistently exhibited higher mortality rates than females. For instance, in 2020, the mortality rate was 8.2% for males compared to 3.8% for females. Similarly, in 2021, the rates were 8.1% for males and 4.4% for females. This disparity in mortality rates may indicate differences in case severity or comorbidities between genders. Mortality rates increased significantly with age, particularly in patients aged 60 years and above. In contrast, among the younger age groups (such as those under 1 year and those between 1 and 4 years), the mortality rates remained relatively low but consistent. Additionally, mortality rates rose with longer hospital stays. Overall, the general mortality rates were consistently low, ranging from 0.3% in 2022 to 0.9% in 2020, while ICU mortality rates were significantly higher than those in general hospital beds.

Table 4 displays the distribution of inpatient causes of death during the study period (2018–2022) categorized by ICD-10 classification. The disease groups with the highest prevalence of mortality include conditions originating in the perinatal period (P00-P96), at 15%, followed by diseases of the circulatory system (I00-I99), at 12%, and diseases categorized under codes for special purposes (U00-U85), also at 12%. Notably, the latter group experienced a significant increase in 2020, rising to 26%, likely attributable to COVID-19.

Table 5 presents the results of the logistic regression analysis examining the risk factors associated with hospital mortality. The analysis indicates that males had an increased mortality risk, with an odds ratio (OR) of 2.3 and a 95% confidence interval (CI) of 2.01 to 2.43. Patients in general bed departments experienced a significantly lower mortality risk compared to those in intensive care units, with an OR of 0.015 and a 95% CI of 0.014 to 0.017. Additionally, the hospital length of stay was correlated with mortality risk. Compared to short stays, medium-length stays were associated with a 70% increased risk of hospital death, reflected by an OR of 1.7 and a 95% CI of 1.53 to 1.94. Extended stays further elevate this risk, with an OR of 5.75 and a 95% CI of 5.19 to 6.36, indicating a 5.75 times higher risk of hospital mortality.

**Figure 1:**
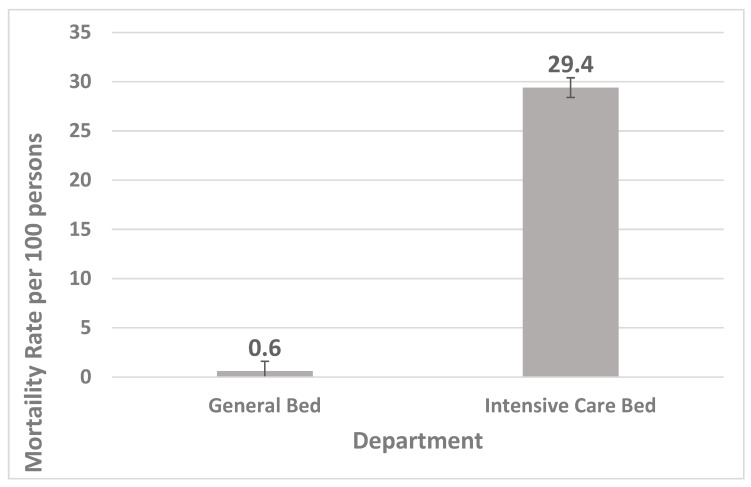
Hospital mortality rate per 100 hospitalized persons for the general bed department compared to the intensive care unit during the period 2018–2022.

Figure 1 compares the hospital mortality rates per 100 hospitalized patients in the general departments and the intensive care unit (ICU) during the period from 2018 to 2022. A significant variation in mortality rates is documented, with general beds reporting 0.6 deaths per 100 patients (95% CI: 0.6–0.7) and the ICU reporting 29.4 deaths per 100 patients (95% CI: 28.5–30.4).

**Figure 2:**
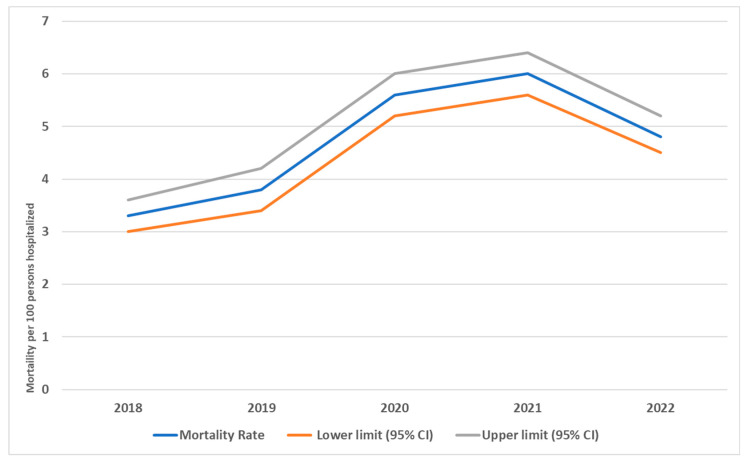
Trend in hospital mortality rate per 100 hospitalized persons during the period 2018–2022.

Figure 2 illustrates the trend in the hospital mortality rate per 100 hospitalized patients during the period from 2018 to 2022 with 95% CI. The graph shows that the trend is low at the beginning of the period, with an average of 3.3 deaths per 100 hospitalized patients. The rate increases from 2018 to 2020, followed by a drop to an average of 4.8 deaths per 100 hospitalized patients in 2022.

**Figure 3:**
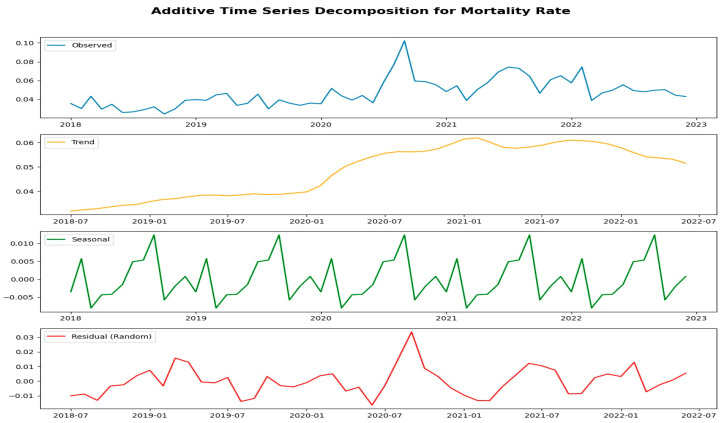
Decomposition of additive time series: hospital mortality rate (2018–2022).

Decomposing a multiplicative time series involves breaking the series down into its three components: trend (T), seasonal (S), and irregular (also known as residual, R). This approach assumes that the relationship among these components is multiplicative. Analyzing the hospital mortality data using the Dickey–Fuller test and the Kwiatkowski–Phillips–Schmidt–Shin (KPSS) test indicates that the data is stationary, as the alpha value is less than 0.05. Furthermore, the decomposition of the multiplicative time series reveals the observed series, the smoothed trend line, the seasonal pattern, and the random component of the series. The overall trend shows an increase, with a slight rise noted at the end of the series (Figure 3).

## 4. Discussion

This retrospective analysis examined the patterns and trends in inpatient mortality at King Fahad Central Hospital, southwest Saudi Arabia, during the period (2018–2022). In addition, we aimed to investigate the characteristics of inpatient mortality, including age-specific mortality rates, length of stay, and other related factors. Studying hospital mortality is essential for understanding and improving healthcare systems, as it provides valuable insights into the quality of care, patient outcomes, and system performance.

The data provide a detailed breakdown of inpatient mortality over the five-year period studied according to characteristics such as sex, age, length of stay (LOS), and department. Annual mortality rates varied, with a peak observed in 2021. The overall mortality rate was the highest in the year 2021, at 6.0%. In contrast, the lowest death rate (3.3%) was observed in 2018. The data reveals that the number of female patients was consistently higher than that of males, regardless of age group, averaging 59.1% for women and 40.9% for men. This gender gap was evident in every year under study, with women always outnumbering men. The mortality rate of male patients was consistently higher than that of female patients during the study period. This trend continued over the years, with the highest male mortality observed in 2020, at 8.2% (407 deaths). COVID-19 severity and mortality are higher in men than they are in women. In a meta-analysis, Rushovich T. (2021) found that men had higher mortality rates from COVID-19 across multiple countries [13].

The data showed significantly higher mortality levels in the hospital during the COVID-19 period. Generally, the COVID-19 pandemic significantly increased hospital mortality rates, both directly due to the disease and indirectly due to overwhelmed healthcare systems and delayed care [14,15]. While pre-pandemic mortality rates were relatively stable, the pandemic exposed vulnerabilities in healthcare systems worldwide, leading to a sharp rise in deaths. After COVID-19, hospital mortality rates have improved compared to the peak of the pandemic but remain higher than pre-pandemic levels in many regions. The lingering effects of the pandemic—such as long COVID, healthcare system strain, and backlogs in care—continue to impact outcomes. In the U.S., excess mortality declined after 2021 but remained higher than pre-COVID-19 baselines due to the indirect effects of the pandemic [16].

Our dataset shows that male mortality increased significantly, at 7.0% (1790 deaths), while female mortality was 3.2% (1195 deaths). The difference in mortality rates between men and women was statistically significant, with a *p*-value < 0.001 and an effect size of 0.09. This trend aligns with findings from a previous systematic review and meta-analysis, which reported higher death rates among males compared to females [17,18]. The study further showed a significantly higher risk of death for male patients compared to females. The crude odds ratio (COR) for male patients was 2.26 (95% CI: 2.09–2.43), with a *p*-value of <0.001, making it statistically significant [17,18].

Mortality patterns vary by age group. Importantly, the highest mortality rate, at 17.2% (1029 deaths), was seen in patients aged 60 years and older. Many studies consistently show that individuals aged 65 years and above, particularly those over 85 years, experienced the highest mortality rates due to COVID-19. This can be attributed to higher susceptibility to severe disease and complications among elderly patients [18]. A recent global study indicated that, during the COVID-19 pandemic, in 2020 and 2021, the death rate for adults around the world rose sharply, going against a previous trend of declining rates [17].

The age group with the lowest mortality was the 15–29-year-old group, at 1.9% (267 deaths). The differences in age-specific mortality between the groups were statistically significant (*p* < 0.001), with a large effect (0.21). Patients aged 5–44 years had an increased risk of mortality compared to patients younger than 5 years, with a COR of 0.49 (95% CI: 0.44–0.54) and a *p*-value of <0.001. Patients aged 45 years and older had a significantly increased risk of mortality, with a COR of 3.45 (95% CI: 3.15–3.76) and a *p*-value of <0.001. In addition, among adults aged 35–64 years, moderate rises in mortality rates were noted; these are frequently associated with pre-existing health conditions and workplace exposure risks. In contrast, children and young adults experienced minimal direct impact, with only slight increases in mortality rates [18,19].

Long-stay patients had the highest mortality rate at 10.3% (1852 deaths), followed by intermediate-stay patients at 3.3% (652 deaths), and short-stay patients at 2.0% (492 deaths). Medium-stay patients had a higher risk of death compared to short-stay patients (COR 1.73 (95% CI: 1.53–1.94), *p*-value < 0.001). Patients with a longer hospital stay had an even higher risk (COR 5.75 (95% CI: 5.19–6.36), *p*-value < 0.001). Prolonged hospital stays are thus associated with a mortality rate more than twice that of patients with shorter stays [19].

Patients in the intensive care unit (ICU) had a significantly higher risk of death compared to those in general departments, with a COR of 0.015 (95% CI: 0.014–0.017) and a *p*-value of <0.001. This highlights the critical condition of patients admitted to the ICU, who are at a much higher risk of mortality. Public health officials and hospital administrators may seek to prevent high COVID-19 ICU demand to optimize outcomes for patients with COVID-19. This conclusion is consistent with those in many previous published reports [19,20,21]. Numerous studies have shown that extended hospital stays are linked to an increased risk of death [19,22].

Some of this study’s implications are worth highlighting. The overall low mortality rate (4.8%) suggests generally effective hospital care, but the higher mortality in males (7.0% vs. 3.2%) signals a need for gender-specific interventions. Moreover, the higher mortality in older adults aligns with global trends but may require targeted geriatric care protocols. This study provides valuable baseline data on hospital mortality trends in Jazan, Saudi Arabia; however, it also highlights critical gaps, evidenced by the high mortality rates of males compared to females. Case-control or cohort studies comparing male vs. female patients with similar conditions may provide in-depth analyses of male-specific mortality risks. Another important issue is the high rates of ICU mortality. The question of how ICU outcomes can be improved is an important one and needs further investigation. Studies focusing on mortality drivers in the ICU vs. general wards using detailed chart reviews of ICU vs. non-ICU deaths may provide an in-depth explanation of the high ICU mortality seen in our study.

This research has several limitations. First, we employed a retrospective study design. Second, this study was conducted at a single tertiary center; the limited population may have skewed the results, contributing to a higher mortality rate and affecting the generalizability of the findings. The reliability of the results would be improved by multicenter studies with larger sample sizes. Finally, the causes of death in this study were initially grouped according to the ICD-10 classification system. However, it is important to highlight that this data utilized the primary diagnosis noted at admission, and not every record had comprehensive or conclusive data, which would facilitate a more accurate cause-of-death classification. Future studies could also consider incorporating more clinical variables related to the risk of hospital mortality.

## 5. Conclusions

This analysis of hospital data statistics revealed a relatively low hospital mortality rate in Jazan. However, the high ICU mortality rates require further analysis and investigation. The results provide a significant insight into the burden of disease and help identify opportunities for systemic improvements. The development of customized interventions targeting high-mortality diseases is recommended. It is also important to explore why male patients have significantly higher mortality rates in order to develop tailored interventions.

## Figures and Tables

**Table 1:** Demographic characteristics of patients according to year of admission (n = 62,534).

Characteristic		Year of Admission										All Years	
		2018		2019		2020		2021		2022			
		N	%	N	%	N	%	N	%	N	%	N	%
Gender	Male	4339	(37.9)	3607	(35.6)	4938	(41.1)	6013	(43.2)	6646	(44.3)	25,543	(40.9)
	Female	7109	(62.1)	6516	(64.4)	7068	(58.9)	7918	(56.8)	8360	(55.7)	36,971	(59.1)
Age groups	<1 year	3428	(29.9)	2978	(29.4)	3173	(26.4)	3278	(23.5)	3271	(21.8)	16,128	(25.8)
	1–4 years	588	(5.1)	433	(4.3)	586	(4.9)	793	(5.7)	1089	(7.3)	3489	(5.6)
	5–14 years	825	(7.2)	687	(6.8)	835	(7.0)	1130	(8.1)	1509	(10.1)	4986	(8.0)
	15–29 years	2953	(25.8)	2536	(25.0)	2710	(22.6)	2888	(20.7)	3069	(20.4)	14,156	(22.6)
	30–44 years	2442	(21.3)	2353	(23.2)	2662	(22.2)	2987	(21.4)	3237	(21.6)	13,681	(21.9)
	45–59 years	526	(4.6)	507	(5.0)	836	(7.0)	1140	(8.2)	1113	(7.4)	4122	(6.6)
	60 + Years	688	(6.0)	633	(6.3)	1207	(10.1)	1719	(12.3)	1725	(11.5)	5972	(9.6)
Nationality	Saudi	8950	(78.2)	7736	(76.4)	9495	(79.1)	11,349	(81.4)	12,308	(82.0)	49,838	(79.7)
	Non-Saudi	2500	(21.8)	2391	(23.6)	2514	(20.9)	2586	(18.6)	2705	(18.0)	12,696	(20.3)
Department	General Bed	10,252	(89.5)	9021	(89.1)	10,142	(84.5)	11,588	(83.2)	12,496	(83.2)	53,499	(85.6)
	ICU	1198	(10.5)	1106	(10.9)	1867	(15.5)	2347	(16.8)	2517	(16.8)	9035	(14.4)
Descriptive measures	Measure	Mean	SD	Mean	SD	Mean	SD	Mean	SD	Mean	SD	Mean	SD
	Age	21.3	20.6	22.2	20.8	25.1	22.9	26.9	24.2	26.2	23.7	24.6	22.8
	LOS	7.8	24.6	8.1	21.4	7.7	18.2	7.5	14.8	6.6	10.6	7.5	18.0
All patients	N	11,450		10,127		12,009		13,935		15,013		62,534	

**Table 2:** Inpatient hospital deaths during the period 2018–2022 according to selected characteristics (n = 62,534).

Characteristic		Rate Per 100 Persons Hospitalized				*p*-Value	Effect Size Phi *φ*/Cramer’s *V*
		Deaths	Mortality Rate	95% CI			
				Lower Limit	Upper Limit		
Gender	Male	1790	7.0	6.7	7.3	<0.001	0.09
	Female	1195	3.2	3.1	3.4		
Nationality	Saudi	2137	4.3	4.1	4.5	<0.001	0.04
	Non-Saudi	859	6.8	6.3	7.2		
Age groups	Less than 1 year	757	4.7	4.4	5.0	<0.001	0.21
	1–4 years	114	3.3	2.7	3.9		
	5–14 years	132	2.6	2.2	3.1		
	15–29 years	267	1.9	1.7	2.1		
	30–44 years	333	2.4	2.2	2.7		
	45–59 years	364	8.8	8.0	9.7		
	60 years and above	1029	17.2	16.3	18.2		
LOS	Short stay	492	2.0	1.8	2.1	<0.001	0.17
	Medium stay	652	3.3	3.1	3.6		
	Long stays	1852	10.3	9.9	10.8		
Department	General bed	338	0.6	0.6	0.7	<0.001	0.47
	Intensive care bed	2658	29.4	28.5	30.4		
Years	2018	377	3.3	3.0	3.6	<0.001	0.05
	2019	385	3.8	3.4	4.2		
	2020	675	5.6	5.2	6.0		
	2021	833	6.0	5.6	6.4		
	2022	726	4.8	4.5	5.2		
Overall		2996	4.8	4.6	5.0		

**Table 3:** Inpatient hospital deaths during the study period according to selected variables.

Years		Gender		Age Group							LOS			Department	
		Male	Female	Less than 1 Year	1–4 Years	5–14 Years	15–29 Years	30–44 Years	45–59 Years	60 Years and Above	Short Stay	Medium Stay	Long Stays	General Bed	Intensive Care Bed
2018	N	218	157	134	13	21	35	36	38	100	64	88	225	75	302
	%	5.0	2.2	3.9	2.2	2.5	1.2	1.5	7.2	14.5	1.3	2.5	7.1	0.7	25.2
	Lower CI	4.4	1.9	3.3	1.2	1.6	0.8	1.1	5.2	12.1	1.0	2.0	6.3	0.6	22.8
	Upper CI	5.7	2.6	4.6	3.6	3.8	1.6	2.0	9.7	17.3	1.7	3.1	8.0	0.9	27.7
2019	N	236	147	130	25	24	35	41	42	88	49	102	234	79	306
	%	6.5	2.3	4.4	5.8	3.5	1.4	1.7	8.3	13.9	1.2	3.3	8.1	0.9	27.7
	Lower CI	5.8	1.9	3.7	3.9	2.3	1.0	1.3	6.1	11.4	0.9	2.7	7.2	0.7	25.1
	Upper CI	7.4	2.6	5.1	8.3	5.1	1.9	2.3	10.9	16.8	1.5	4.0	9.2	1.1	30.4
2020	N	407	266	155	22	23	44	71	105	255	110	149	416	89	586
	%	8.2	3.8	4.9	3.8	2.8	1.6	2.7	12.6	21.1	2.2	4.4	11.8	0.9	31.4
	Lower CI	7.5	3.3	4.2	2.4	1.8	1.2	2.1	10.4	18.9	1.8	3.7	10.7	0.7	29.3
	Upper CI	9.0	4.2	5.7	5.5	4.0	2.2	3.3	14.9	23.5	2.6	5.1	12.9	1.1	33.5
2021	N	486	346	199	24	24	69	90	100	327	141	146	546	63	770
	%	8.1	4.4	6.1	3.0	2.1	2.4	3.0	8.8	19.0	2.5	3.5	13.2	0.5	32.8
	Lower CI	7.4	3.9	5.3	2.0	1.4	1.9	2.4	7.2	17.2	2.1	2.9	12.2	0.4	30.9
	Upper CI	8.8	4.8	6.9	4.4	3.1	3.0	3.7	10.5	20.9	3.0	4.0	14.3	0.7	34.7
2022	N	443	279	139	30	40	84	95	79	259	128	167	431	32	694
	%	6.7	3.3	4.2	2.8	2.7	2.7	2.9	7.1	15.0	2.3	3.2	10.1	0.3	27.6
	Lower CI	6.1	3.0	3.6	1.9	1.9	2.2	2.4	5.7	13.4	2.0	2.7	9.3	0.2	25.9
	Upper CI	7.3	3.7	5.0	3.9	3.6	3.4	3.6	8.7	16.8	2.8	3.7	11.1	0.4	29.3

**Table 4:** Inpatient hospital causes of death during the study period categorized by ICD-10 classification.

Group of Disease	Years					Total
	2018	2019	2020	2021	2022	
Certain conditions originating in the perinatal period (P00-P96)	18%	21%	13%	15%	11%	15%
Certain infectious and parasitic diseases (A00-B99)	11%	13%	9%	5%	5%	8%
Codes for special purposes (U00-U85)	0%	0%	26%	19%	4%	12%
Congenital malformations, deformations, and chromosomal abnormalities (Q00-Q99)	7%	3%	2%	2%	3%	3%
Diseases of the blood and blood-forming organs and certain disorders involving the immune mechanism (D50-D89)	1%	1%	1%	1%	2%	1%
Diseases of the circulatory system (I00-I99)	10%	11%	9%	11%	17%	12%
Diseases of the digestive system (K00-K93)	9%	8%	6%	6%	5%	6%
Diseases of the genitourinary system (N00-N99)	7%	7%	7%	7%	7%	7%
Diseases of the musculoskeletal system and connective tissue (M00-M99)	1%	1%	0%	0%	0%	0%
Diseases of the nervous system (G00-G99)	2%	3%	2%	3%	3%	3%
Diseases of the respiratory system (J00-J99)	7%	9%	6%	5%	9%	7%
Diseases of the skin and subcutaneous tissue (l00-l99)	1%	0%	0%	0%	0%	0%
Endocrine, nutritional, and metabolic diseases (E00-E90)	2%	2%	2%	1%	2%	1%
Facexternal causes of morbidity and mortality (V01-Y98)	0%	2%	2%	1%	3%	2%
Factors influencing health status and contact with health services (Z00-Z99)	2%	2%	1%	2%	2%	2%
Injury, poisoning, and certain other consequences of external causes (S00-T98)	14%	11%	6%	11%	15%	11%
Mental and behavioral disorders (F00-F99)	0%	0%	0%	0%	0%	0%
Neoplasms (C00-D48)	2%	2%	1%	2%	1%	1%
Pregnancy, childbirth, and the puerperium (O00-O99)	1%	1%	1%	0%	1%	1%
Symptoms, signs, and abnormal clinical and laboratory findings, not elsewhere classified (R00-R99)	3%	4%	4%	7%	10%	6%
Missing and unclassified	2%	2%	2%	1%	0%	1%
Total	100%	100%	100%	100%	100%	100%

**Table 5:** Univariate logistic regression analysis of the risk factors associated with hospital deaths.

Factors	*p*-Value	COR	95% CI	
			Lower	Upper
Gender				
Female		1		
Male	<0.001	2.26	2.09	2.43
Age groups				
Less than 5 years		1		
5–44 years	<0.001	0.49	0.44	0.54
45 years and more	<0.001	3.45	3.15	3.76
Department				
General bed				
Intensive care bed	<0.001	0.015	0.014	0.017
Length of stay in days				
Short stay		1		
Medium stay	<0.001	1.73	1.53	1.94
Long stay	<0.001	5.75	5.19	6.36

COR = crude odds ratio; 95% CI = 95% confidence interval.

## Data Availability

The datasets used and/or analyzed during the current study are available from the corresponding author (MSM) upon reasonable request.

## Abbreviations

The following abbreviations are used in this manuscript:

ICU	intensive care unit
LOS	length of stay
CI	confidence interval
ICD	International Classification of Diseases
KFCH	King Fahad Central Hospital
